# Dropped Head Syndrome Treated with Physical Therapy Based on the Concept of Athletic Rehabilitation

**DOI:** 10.1155/2020/8811148

**Published:** 2020-12-08

**Authors:** Toshio Mori, Kentaro Mataki, Yukiyo Shimizu, Kai Matsuba, Kosei Miura, Hiroshi Takahashi, Masao Koda, Hiroshi Kamada, Masashi Yamazaki

**Affiliations:** ^1^Center for Sports Medicine and Health Science, University of Tsukuba Hospital, 1-1-1 Tennodai, Tsukuba, Ibaraki 305-8575, Japan; ^2^Department of Orthopaedic Surgery, Faculty of Medicine, University of Tsukuba, 1-1-1 Tennodai, Tsukuba, Ibaraki 305-8575, Japan; ^3^Department of Rehabilitation Medicine, Faculty of Medicine, University of Tsukuba, 1-1-1 Tennodai, Tsukuba, Ibaraki 305-8575, Japan

## Abstract

Patients with dropped head syndrome (DHS) show severe cervical kyphosis, i.e., chin-on-chest deformity, and their activities of daily living are impaired considerably. However, the therapeutics for DHS, especially conservative treatment, have not been fully established. A 75-year-old woman suffered from DHS, which she developed from neck pain due to cervical spondylosis. Examinations showed atrophy and dysfunction of her cervical extensor muscles. For this patient, we created a special program of physical therapy based on the concept of athletic rehabilitation and provided her the athletic rehabilitation-based physical therapy (AR-PT). After starting AR-PT, the patient's neck pain was relieved. She recovered from DHS, and the atrophy of her cervical extensor muscles improved. This study suggests that our program of AR-PT improves cervical extensor muscle insufficiency in patients with DHS and corrects their cervical kyphosis.

## 1. Introduction

Patients with dropped head syndrome (DHS) show severe cervical kyphosis, i.e., chin-on-chest deformity. They cannot preserve forward vision, and their activities of daily living (ADL) are impaired considerably [1, 2]. DHS develops from several etiologies, including extrapyramidal tract disease, e.g., Parkinson's disease, motor neuron disease, neuromuscular diseases such as myasthenia gravis and myositis, spine diseases such as cervical spondylotic myelopathy and cervical spondylotic amyotrophy, and conditions following cervical spine surgery [1–5]. Recently, we often encounter elderly DHS patients with sarcopenia [6]. Maki et al. previously reported a patient with DHS due to lower leg pain, though this is a very rare condition [7]. As DHS results from many different diseases, a variety of mechanisms seem to be involved in its development. When we treat patients with DHS, first we should evaluate the mechanism that causes DHS in that patient and then select an appropriate therapeutic according to the specific pathology [8].

Previous reports have described pharmacotherapy, surgery, and rehabilitation as therapeutic treatments for DHS [1–4, 9]. The primary rehabilitation procedures for DHS have been strengthening exercises of the cervical extensor muscles [10, 11]. However, there have been few reports that describe the exercises in detail, and the outcomes of such rehabilitation therapy are still unclear.

In this report, we present a patient with DHS, in whom DHS developed originating from neck pain due to cervical spondylosis. For this patient, we created a special program of physical therapy based on the concept of athletic rehabilitation and provided her with the athletic rehabilitation-based physical therapy (AR-PT) [12, 13]. After starting the AR-PT, the patient's neck pain was relieved and her DHS also was improved.

## 2. Case Presentation

A 75-year-old Asian woman suffered from neck pain and restricted neck extension without any trauma. Initially, she felt no pain when lying down, but neck pain appeared when she was standing. Thereafter, her neck pain worsened and DHS appeared, so she visited the outpatient clinic of our hospital.

**Figure 1:**
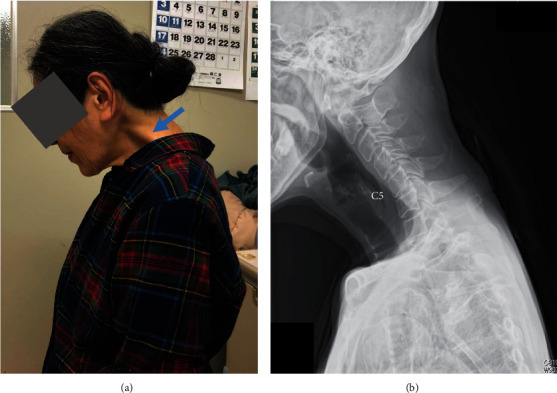
Lateral view of the patient's neck (a) and her cervical lateral radiograph (b) at the first visit. (a) Dropped head syndrome (DHS) was evident. Remarkable bulging was seen over her trapezius and levator scapulae muscles (a, arrow). (b) The patient's cervical spine inclined forward, and kyphotic deformity was present at C5-C6.

At the first visit, her DHS posture was evident, and her neck extension was severely restricted (Figure 1(a)). Remarkable bulging was seen over her trapezius and levator scapulae muscles (Figure 1(a), arrow). There were no signs of myelopathy such as motor paralysis of her upper or lower extremities or increased deep tendon reflexes. Her DHS carriage became more evident when she walked. Cervical lateral radiographs showed that her cervical spine inclined forward, and kyphotic deformity was present at C5-C6 (Figure 1(b)). Measurement of her cervical spine alignment revealed that the center of gravity of the head- (CGH-) C7 sagittal vertical axis (SVA) was 67 mm, C2-C7 SVA was 44 mm, cervical lordosis (CL) was 32 degrees, and T1 slope (T1S) was 23 degrees.

**Figure 2:**
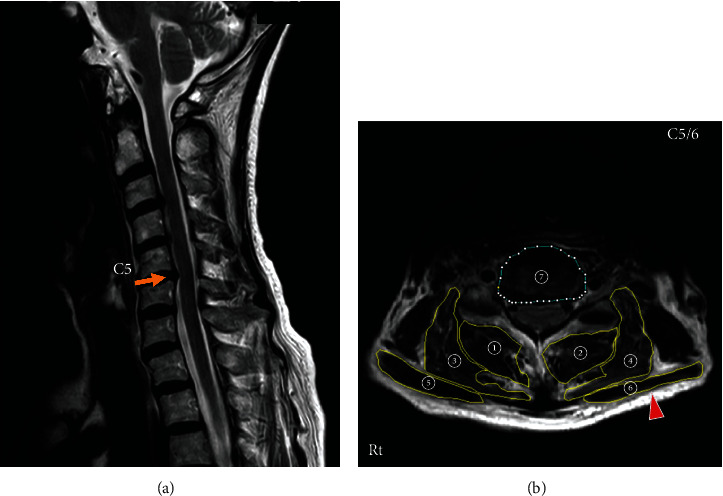
T2-weighted magnetic resonance (MR) sagittal image (a) and axial image at C5-C6 (b) of the patient's cervical spine at the first visit. (a) Cervical alignment was slightly kyphotic, and the C5-C6 intervertebral disc protruded posteriorly (arrow). (b) The cervical extensor muscles were atrophic. The trapezius muscles showed a stretched morphology (arrowhead). ➀➁: deep layers of the cervical extensor muscles (semispinalis cervicis and multifidus). ➂➃: lateral-dorsal layers of the cervical extensor muscles (splenius capitis, splenius cervicis, and longissimus). ➄➅: trapezius. ➆: C5-C6 disc.

Cervical magnetic resonance (MR) images showed that her cervical alignment was slightly kyphotic, and the C5-C6 intervertebral disc protruded posteriorly (Figure 2(a), arrow). An axial view MRI at C5-C6 showed atrophy of cervical extensor muscles (Figure 2(b)). The cross-sectional area of the cervical posterior muscles at C5-C6 was measured on the axial view MRI according to the method of Okada et al. [14]. The cross-sectional area of the deep layers of the cervical extensor muscles (semispinalis cervicis and multifidus) was 265.0 mm^2^, and that of the lateral-dorsal layers of the cervical extensor muscles (splenius capitis, splenius cervicis, and longissimus) was 360.6 mm^2^ (Table 1). These data were smaller than those of asymptomatic volunteers which were reported by Okada et al. [14]. The cross-sectional area of the trapezius muscles was 182.5 mm^2^ (Table 1), and morphologically, her trapezius muscles were stretched (Figure 2(b), arrowhead).

We applied a cervical collar and prescribed analgesic drugs (celecoxib, tramadol-acetaminophen combination). However, no pain relief was obtained, and her DHS posture continued.

**Figure 3:**
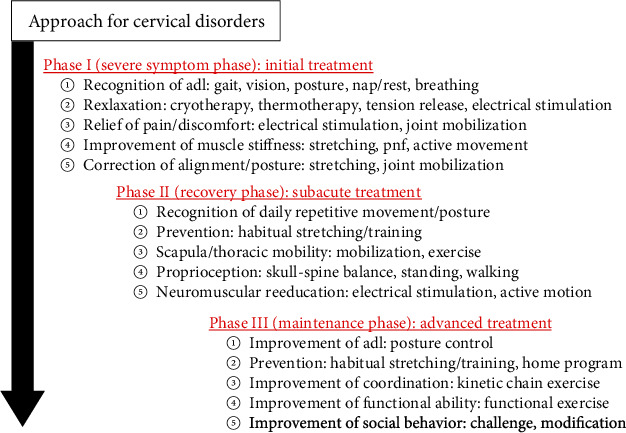
The principle of our approach to cervical disorders in athletes.

**Figure 4:**
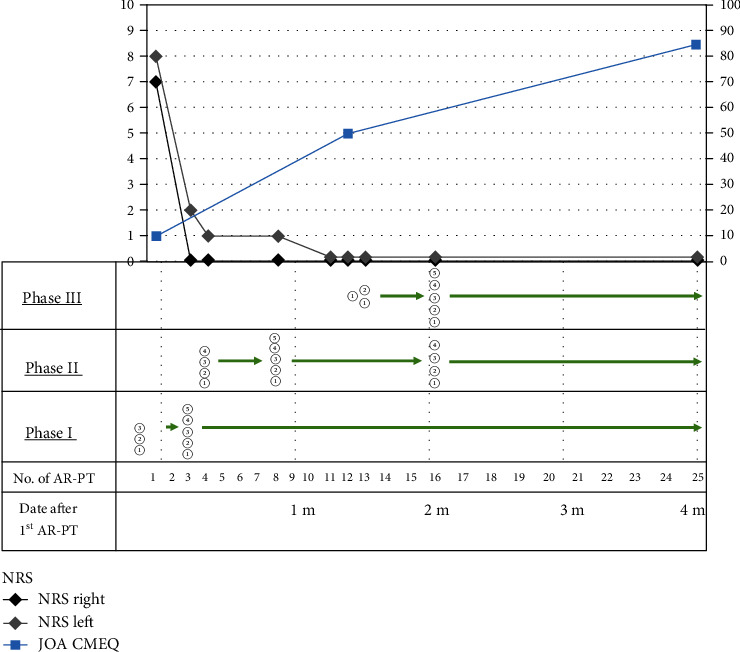
The program of athletic rehabilitation-based physical therapy (AR-PT) for the present patient and the change of her symptoms. Programs applied for this patient are shown. The circled numbers at phase I, phase II, and phase III are the programs described in Figure 3. NRS: Numerical Rating Scale; JOA-CMEQ: Japanese Orthopaedic Association-Cervical Myelopathy Evaluation Questionnaire; m: month.

**Figure 5:**
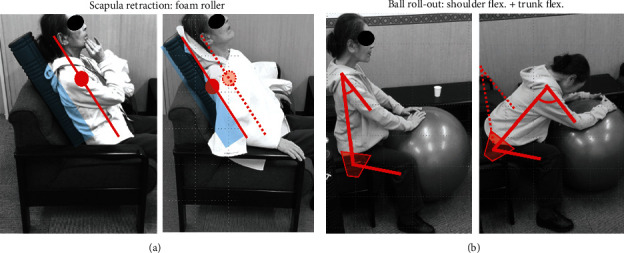
Representative exercises of our AR-PT (phase II) for the present patient. (a) Scapular retraction with a foam roller. As the patient was able to lean back on the sofa only with head support, scapular retraction was introduced for her to become aware of her habitual control of posture. This exercise corresponded to the phase II ➀➁➂ programs. (b) Ball roll-out exercise for shoulder flexion and trunk flexion. Kinesthetic awareness of craniospinal balance needed to be learned initially, and proprioception and coordination training of her posture were taught such as shoulder flexion, trunk flexion, and anterior pelvic tilt. This exercise corresponded to the phase II ➀➁➂ programs.

**Figure 6:**
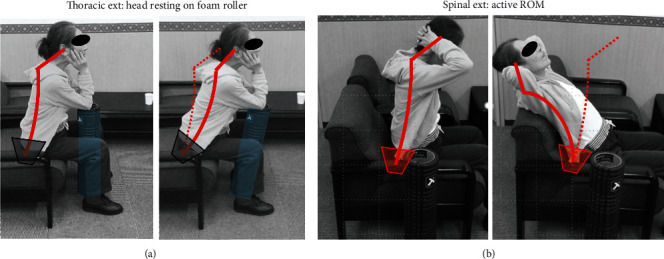
Representative exercises of our AR-PT (phase III) for the present patient. (a) Thoracic extension with head resting on a foam roller. The goal of this exercise was to mobilize and control the cervical and upper thoracic regions while her head was stabilized. The lower thoracic and lumber regions also were consciously controlled with anterior pelvic tilting so that the desired sitting position was under the patient's own control. This exercise corresponded to the phase III ➁➂➃ programs. (b) Spinal extension within the submaximal active range of motion. The goal of this exercise was to activate cervical and upper thoracic regions while her head was stabilized under her own control. Both suboccipital flexion and pelvic anterior tilt were controlled throughout this movement. This exercise corresponded to the phase III ➀➁➂ programs.

Two weeks after the first visit, we began to provide her with AR-PT. Prior to the AR-PT, we evaluated her neck condition and found radiating pain from the neck to shoulder (Numerical Rating Scale (NRS): Rt = 7, Lt = 8), inappropriate spinal alignment from the occipito-cervical to cervico-thoracic area, and excessive abduction of the scapula. The Japanese Orthopaedic Association-Cervical Myelopathy Evaluation Questionnaire (JOA-CMEQ) score at this point was 10, indicating a marked decline of her cervical spine function. According to our approach to cervical disorders in athletes (Figure 3), we provided her with our AR-PT (Figure 4). As an initial treatment, we applied phase I programs for relieving her severe symptoms. Subsequently, we added phase II programs as a subacute treatment and then phase III programs as an advanced treatment. One AR-PT session took 60 minutes, and initially, she underwent AR-PT twice a week. The details of representative exercises of our AR-PT for this patient are shown in Figures 5 and 6.

**Figure 7:**
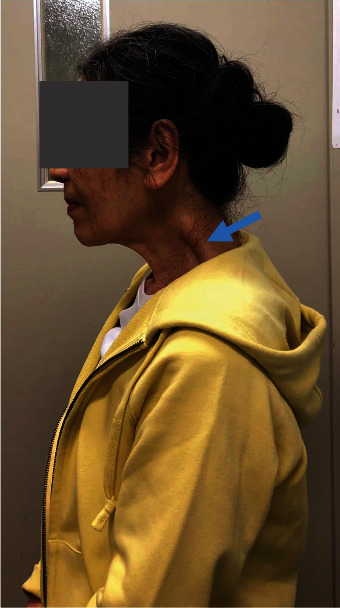
Lateral view of the patient's neck at the 3rd AR-PT. DHS was improved. The bulged muscles were still seen at this point (arrow).

At the third AR-PT, the patient's neck pain was relieved with only slight pain and stiffness on the left side of her neck (NRS: Rt = 0, Lt = 2) (Figure 4). Her cervical extension was improved, and she was able to use a cup to drink and gargle (Figure 7). However, the bulged muscles were still seen at this point (Figure 7, arrow).

At the 12th AR-PT, the patient's neck pain was relieved completely (NRS: Rt = 0, Lt = 0) (Figure 4). She underwent AR-PT twice a week for the initial two months during the recovery phase which was then reduced to once a week for another three months during the maintenance phase. Four months after the first AR-PT, the JOA-CMEQ score increased to 86, indicating a sufficient functional recovery. After five-month AR-PT, she was instructed to continue physical exercise at home.

**Figure 8:**
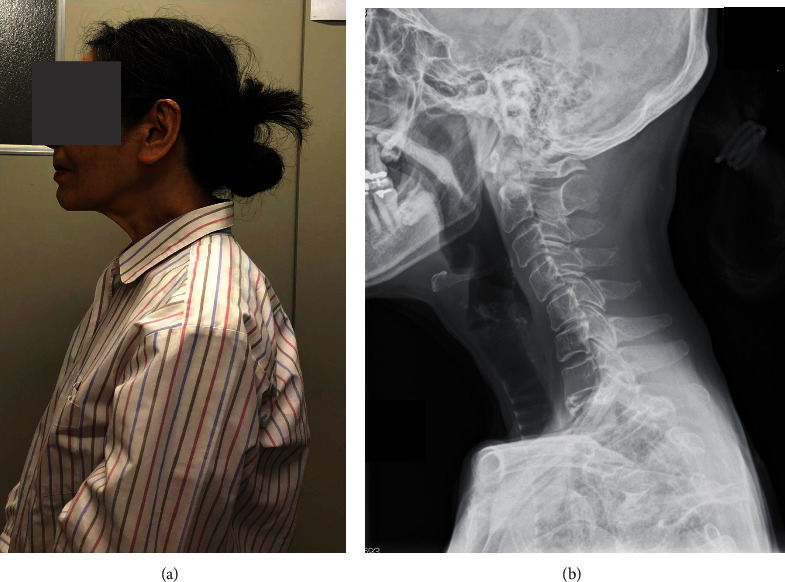
Lateral view of the patient's neck (a) and her cervical lateral radiograph (b) seven months after the 1st AR-PT. (a) The patient's neck posture was normal, and the muscle bulging had disappeared. (b) Forward inclination of her cervical spine was improved.

Seven months after the first AR-PT, her cervical posture became normal, and the muscle bulging disappeared (Figure 8(a)). No DHS appeared while walking. Cervical radiographs showed that forward inclination of her cervical spine was improved (Figure 8(b)). Parameters of her cervical spine alignment were CGH-C7 SVA: 49 mm, C2-C7 SVA: 39 mm, CL: 26 degrees, and T1S: 16 degrees.

**Figure 9:**
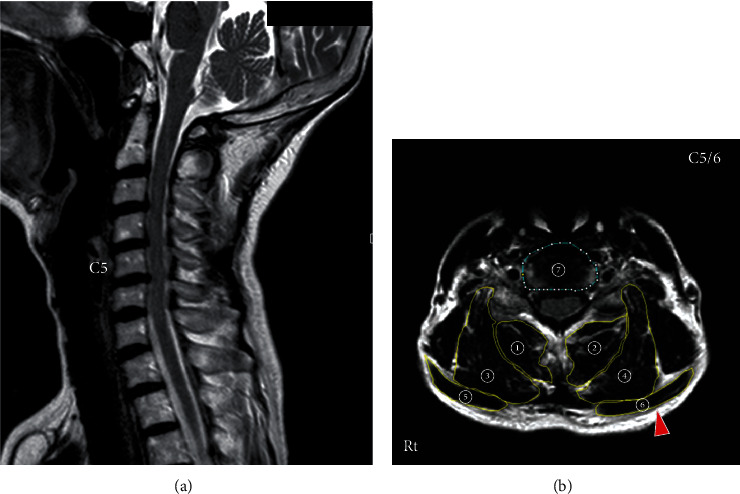
T2-weighted MR sagittal image (a) and axial image at C5-C6 (b) of the patient's cervical spine eight months after the first AR-PT. (a) Alignment of her cervical spine became normal. (b) Atrophy of her cervical extensor muscles was improved. Muscle tonus of the trapezius was restored (arrowhead). ➀➁: deep layers of cervical extensor muscles (semispinalis cervicis and multifidus). ➂➃: lateral-dorsal layers of cervical extensor muscles (splenius capitis, splenius cervicis, and longissimus). ➄➅: trapezius. ➆: C5-C6 disc.

Cervical MR images eight months after the first AR-PT showed that alignment of her cervical spine had become normal and atrophy of her cervical extensor muscles was improved (Figure 9(b)). At the C5-C6 level, the cross-sectional area of the deep layers of cervical extensor muscles was 324.6 mm^2^ and that of the lateral-dorsal layers of the cervical extensor muscles was 615.9 mm^2^, both of which were greater than those before AR-PT. The cross-sectional area of the trapezius muscles was 162.3 mm^2^, which was almost the same as before AR-PT (Table 1). However, their morphology indicated that muscle tonus was restored after AR-PT (Figure 9(b), arrowhead).

Seventeen months after the first AR-PT, her neck posture was normal, indicating no recurrence of DHS.

## 3. Discussion

We suggest that the mechanism for DHS in this patient was a spondylotic change of her cervical spine and especially posterior protrusion of the C5-C6 disc, caused by severe neck pain which suppressed the function of her cervical extensor muscles. The decreased cross-sectional area of her cervical extensor muscles demonstrated the atrophy and dysfunction of those muscles.

In contrast, her trapezius muscles did not show atrophy, and their tonus was increased. This means that despite such severe neck pain, her trapezius muscles maintained their function. We suggest that in this patient, the trapezius muscles helped to prevent further progression of DHS. The accessory nerve innervates the trapezius muscles whereas the cervical extensor muscles are innervated by spinal nerves. We speculate that this difference of innervation is one reason why the trapezius muscles were preserved in this patient.

Rehabilitation has been a major approach as conservative therapy for DHS. Most previous reports recommended strengthening exercises of the cervical extensor muscles [10, 11]. However, there have been few reports that describe either the details or the effects of those exercises. Previously, we applied such muscle strengthening exercises for DHS patients as conventional rehabilitation, but improvement of their DHS was limited. Thus, we realized that conventional rehabilitation for DHS has limitations. For this patient, therefore, we created an original program of physical therapy based on the concept of athletic rehabilitation. As a result of our AR-PT, dramatic improvement of the patient's DHS was achieved.

Athletic rehabilitation is a field in which sports-related injuries and disorders of athletes are treated thoroughly to allow a return to high-level athletic performance [12, 13]. Early pain relief is essential for recovering the function of an injured athlete's body with the least possible delay. Therapists are required to understand the details of the injury, evaluate tissue damage, and maximize efforts to achieve early pain relief. In addition, therapists should analyze the factors that delay the recovery of the athlete's injury and provide them with physical therapy to prevent the aggravation [12, 13].

The principle of athletic rehabilitation is not a local treatment for the injured site but multiple exercises for the whole body including noninjured sites. To accomplish this, therapists must evaluate the joint range of motion, muscle power, and flexibility of the athlete's whole body. In athletic rehabilitation, the importance of training for trunk balance/posture control and neuromuscular reeducation is emphasized. Athletes should feel by themselves that exercise can relieve their pain: this successful experience of exercise-induced pain relief is quite important [12, 13].

In our university hospital, we established the “Center for Sports Medicine and Health Science” in 2015 and have supported injured athletes to return to play under the collaboration of the medical and athletic departments. For treatment of the present DHS patient, we created a specific physical therapy program in this center using the concepts of athletic rehabilitation (Figure 3). The initial treatment for pain relief during the severe symptom phase was quite effective for improving her DHS. Therapists then provided her with several treatments for skull-spine balance training and posture control (Figures 5 and 6). We believe that our programs of AR-PT caused the improvement of DHS in this patient and the maintenance of the therapeutic effects after finishing the program of AR-PT.

Miura et al. previously reported a case of DHS, in which gait training with the extraskeletal robot suit HAL immediately improved the patient's DHS [15]. The target of gait training with HAL is the patient's lower extremities and pelvic region. Thus, it is possible that exercise of the lower extremities and pelvic region positively affects the patient's cervical extensor muscles. Kadone et al. also found that gait training with HAL induced anterior inclination of the patient's pelvis [16]. We speculate that such anterior inclination of the pelvis may contribute to the improvement of DHS. In the present AR-PT, we emphasized skull-spine balance, posture control, and neuromuscular reeducation, and these programs include anterior inclination of the pelvis. Thus, we suggest that the present AR-PT improves DHS similar to the improvement achieved by HAL gait training.

Previously, Ikumi et al. performed HAL gait training for a patient with cervical spinal cord injury whose upper and lower extremities were paralyzed. After the HAL gait training, muscle tonus was decreased not only in the lower extremities but also in the upper extremities [17]. This suggests that motor function of lower extremities, trunk, and upper extremities should not be considered independent. Shimizu et al. also have observed the improvement of lower limb muscle function in patients with complete spinal cord injury after the upper limb-triggered HAL [18]. These findings suggest that a certain synergistic regulation of motor function may exist in humans.

Igawa et al. reported that a localized rehabilitation program such as cervical extensor muscle training alone is insufficient for DHS patients. They showed that their program including thoracic mobilization and anterior pelvic tilt exercises improved DHS [19]. This is consistent with the present data.

Surgical treatment can dramatically correct the cervical kyphosis of DHS patients [4, 9]. After surgery, however, patients' neck mobility is severely restricted. This is a great disadvantage for patients. Thus, surgical indications for DHS should be considered carefully. DHS includes various pathologies, and the mechanism by which it develops is patient-specific. The mechanism of DHS should be evaluated in each patient and the most appropriate therapeutic method selected. For DHS patients who have cervical extensor muscle insufficiency originating from neck pain as in the present case, our program based on the concept of athletic rehabilitation is one option.

## 4. Conclusions

This study suggests that a program of AR-PT is capable of improving cervical extensor muscle insufficiency in patients with DHS and corrects their cervical kyphosis.

## Figures and Tables

**Table 1:** Cross-sectional area of the cervical extensor muscles and trapezius at the C5-C6 level.

	Before AR-PT	After AR-PT
Deep layers (mm^2^)	265.0	324.6
Semispinalis cervicis		
Multifidus		
Lateral-dorsal layers (mm^2^)	360.6	615.9
Splenius capitis		
Splenius cervicis		
Longissimus		
Trapezius (mm^2^)	182.5	162.3

AR-PT: athletic rehabilitation-based physical therapy; after AR-PT: eight months after the first AR-PT.

## Data Availability

No data were used to support this study.
